# The Role of Non-Native Interactions in the Folding of Knotted Proteins

**DOI:** 10.1371/journal.pcbi.1002504

**Published:** 2012-06-14

**Authors:** Tatjana Škrbić, Cristian Micheletti, Pietro Faccioli

**Affiliations:** 1 ECT* - European Centre for Theoretical Studies in Nuclear Physics and Related Areas, Villazzano (Trento), Italy; 2 LISC - Interdisciplinary Laboratory for Computational Science, Povo (Trento), Italy; 3 SISSA - Scuola Internazionale Superiore di Studi Avanzati and CNR-IOM Democritos, Trieste, Italy; 4 Dipartimento di Fisica, Università degli Studi di Trento, Povo (Trento), Italy; 5 INFN - Istituto Nazionale di Fisica Nucleare, Gruppo Collegato di Trento, Povo (Trento), Italy; Iowa State, United States of America

## Abstract

Stochastic simulations of coarse-grained protein models are used to investigate the propensity to form knots in early stages of protein folding. The study is carried out comparatively for two homologous carbamoyltransferases, a natively-knotted N-acetylornithine carbamoyltransferase (AOTCase) and an unknotted ornithine carbamoyltransferase (OTCase). In addition, two different sets of pairwise amino acid interactions are considered: one promoting exclusively native interactions, and the other additionally including non-native quasi-chemical and electrostatic interactions. With the former model neither protein shows a propensity to form knots. With the additional non-native interactions, knotting propensity remains negligible for the natively-unknotted OTCase while for AOTCase it is much enhanced. Analysis of the trajectories suggests that the different entanglement of the two transcarbamylases follows from the tendency of the C-terminal to point away from (for OTCase) or approach and eventually thread (for AOTCase) other regions of partly-folded protein. The analysis of the OTCase/AOTCase pair clarifies that natively-knotted proteins can spontaneously knot during early folding stages and that non-native sequence-dependent interactions are important for promoting and disfavouring early knotting events.

## Author Summary

Knotted proteins provide an ideal ground for examining how amino acid interactions (which are local) can favor their folding into a native state of non-trivial topology (which is a global property). Some of the mechanisms that can aid knot formation are investigated here by comparing coarse-grained folding simulations of two enzymes that are structurally similar, and yet have natively knotted and unknotted states, respectively. In folding simulations that exclusively promote the formation of native contacts, neither protein forms knots. Strikingly, when sequence-dependent non-native interactions between amino acids are introduced, one observes knotting events but only for the natively-knotted protein. The results support the importance of non-native interactions in favoring or disfavoring knotting events in the early stages of folding.

## Introduction

A much debated problem in the thermodynamics and kinetics of protein folding [1], [2] is the process of knot formation in proteins [3], [4], [5], [6], [7], [8]. The topological entanglement found in naturally-occurring proteins presents several differences from that of compact or collapsed flexible polymers [9]. Firstly, the overall percentage of knotted native states in the protein data bank (PDB) is lower than for globular flexible chains with the same degree of polymerization [10], [11], [12], [13], [14], [15], [16], [17], [18], [19]. In addition, the free energy landscape of proteins, unlike that of homopolymers or random heteropolymers, is sufficiently smooth to ensure that the same knot in the same protein location is observed in folded structures [20], [21], [22], [23], [24], [25].

These distinctive features are arguably the consequence of concomitant effects, including the propensity of polypeptides to form secondary structure elements, which enhance their local order compared to a typical collapsed polymer chain [9], as well as functionally-oriented evolutionary mechanisms [26], [27], [28].

To gain insight into such mechanisms as well as to highlight general physico-chemical mechanisms favoring knot formation, an increasing number of experimental and numerical studies of knotted proteins have been carried out [9], [20], [21], [25], [29], [30], [31], [32], [33], [34], [35], [36]. Notably, the latest experimental results have clarified that native knots can form spontaneously and efficiently from an unknotted initial state. In particular, Yeates and coworkers [21] successfully designed a protein which can refold into the natively-knotted structure, albeit much more slowly than an unknotted counterpart. Finally, the recent study of Mallam and Jackson showed that newly translated, knot free, YibK molecules fold spontaneously to the native trefoil-knotted state, and that chaperones can significantly speed up the folding process [20].

Numerical simulations have been a valuable complement to experiments, particularly regarding the characterization of the pathways leading to the self-tying of knotted proteins. In particular, folding simulations based on simplified protein representations and/or force fields, have indicated two main mechanisms leading to knot formation: the threading of one of the termini through a loop [35], or by slipping a pseudoknot through a loop [36]. The two mechanisms are not mutually exclusive, as reported by Sulkowska *et al.* [25] for protein MJ0366 from *Methanocaldococcus jannaschii*. Importantly, in coarse-grained (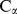-trace) simulations where folding was promoted by exclusively favoring native contacts it was seen that the yield of folding trajectories was low and knots would form at late folding stages. Specifically, for the deeply-knotted protein YibK, only 1–2% of the trajectories reached the native state and the native knot was formed when about 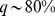 of the native contacts had already been established. Arguably, the coarse-grained nature of the models contributes to the observed low folding yield. However, even when employing atomistic representations, purely native-centric models tend to favor a rather late onset of knotting [37]. For example, a relatively late stage of knot formation 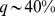 was also estimated from the analysis of the free-energy landscape of protein MJ0366, despite being half as long as YibK and with a shallower knot [25].

These findings are aptly complemented by the early observation of Wallin *et al.* [35] that the low yield of purely native-centric models can be dramatically enhanced by promoting the attraction of specific protein regions that are not in contact in the native state. In particular, it was seen that the formation of the native knot in YibK was particularly facilitated by introducing an *ad hoc* non-native attraction between a loop and the C-terminus that, by threading the former, produced a sizeable population of knots even at early folding stages, 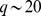
% [35].

These results, together with recent experimental ones [20], [21], [34], pose the question of whether natively-knotted proteins can form non-trivial entanglements during early folding stages and whether non-native interactions can provide a general mechanisms for such self-tying events. Clarifying such aspects is important to advance the understanding of some of the general mechanisms aiding knot formation. It must however, be borne in mind that there could co-exist independent pathways where knots are established at different stages of the folding process and that active mechanisms, such as interactions with chaperones, could be involved *in vivo* [20].

**Figure 1:**
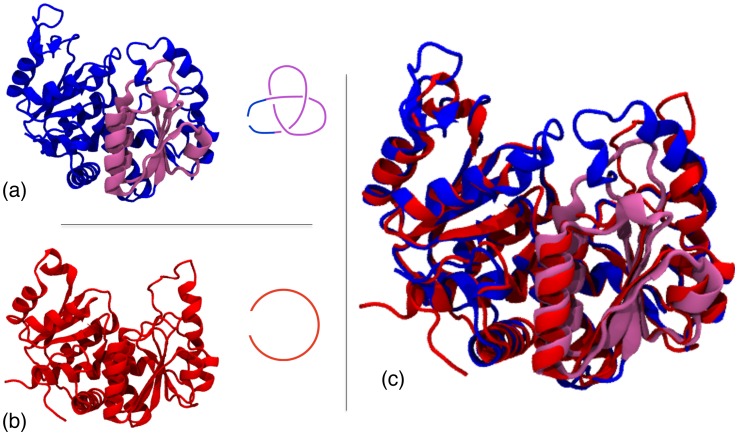
Knotted and unknotted carbamoyltransferases. (a) Cartoon representation of the AOTCase which is natively-knotted in a right-handed trefoil knot (see sketch). The knotted region is highlighted in purple. (b) Cartoon representation of the unknotted OTCase. The MISTRAL structural alignment [39] of the knotted AOTCase and unknotted OTCase is shown in panel (c).

In this study we address these questions by simulating the early folding process of two transcarbamylase proteins that are structurally very similar and yet their native states are differently knotted [26], [27]. Specifically, one of them is a trefoil-knotted N-acetylornithine carbamoyltransferase (AOTCase), while the other is an unknotted ornithine carbamoyltransferase (OTCase), see Fig. 1. Their evolutionary relatedness has posed the interesting question of understanding the source of their different native topology, with particular regards to the role of specific loop regions whose “virtual” excision or addition alters the native topology [27].

Here, for both the OTCase and AOTCase, more than a hundred folding simulations are carried out using a coarse-grained model and two different energy functions. We first evolve a fully extended configuration and steer it towards the native state by exclusively promoting native contact interactions. In a second set of simulations we enrich the energy function by adding quasi-chemical and electrostatic non-native interactions. In both cases, the stochastic evolution is followed up to the formation of about 35% of the native contacts and the knotted topology of the partially-folded structures is monitored.

We find that knot occurrence is negligible for both the AOTCase and OTCase when the energy function favoring only native contacts is used. A dramatic difference in knotting propensity is instead seen when the quasi-chemical non-native interactions are added. In this case, the level of self-entanglement remains negligible for the OTCase but is greatly enhanced for the AOTCase, which is natively knotted. Notably, all proper and improper knots formed in these early folding stages have the correct (native) trefoil topology and chirality.

The analysis of the repeated knotting/unknotting events observed in the simulated trajectories indicates that knotting usually results from the threading of the C-terminal through loops present in the loose protein globule. The threading events are favored by the effective (non-native) attraction of the C-terminus to other protein regions. Additional folding simulations carried out for “in silico mutants” of the AOTCases, provide further support for the effect of the C-terminus chemical composition on the knotting propensities.

## Results/Discussion

The present investigation of knot formation in proteins that are only partially folded is focused on two transcarbamylases: AOTCase, PDB entry 2g68, and OTCase, PDB entry 1pvv. The monomeric units of these two proteins have nearly the same length (332 and 313 amino acids, respectively) and are evolutionarily related. This is established from their significant sequence homology (same CATH code, 3.40.50.1370 [38] and sequence identity equal to 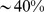). Despite their statistically-significant structural alignability (Mistral p-value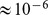 [27], [39]) they have a different knotted topology [26], [27], see Fig. 1. Specifically, the OTCase is unknotted, while the AOTCase contains a right-handed trefoil knot. The knotted region, established using the algorithm of Ref. [40], corresponds to the K172-G255 segment and is at a distance of about 80 amino acids from the C terminus, which is the nearest one in sequence. Because the biological units of the two proteins have a different oligomeric state it is assumed that, consistently with other multimeric knotted proteins, the folding of the monomeric units precedes their assembly. We shall therefore limit considerations to the monomeric units.

### The model

For the purpose of our study, both proteins are described with a simplified structural model, where each amino acid is represented by one interaction center, coinciding with the 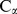 atom.

**Figure 2:**
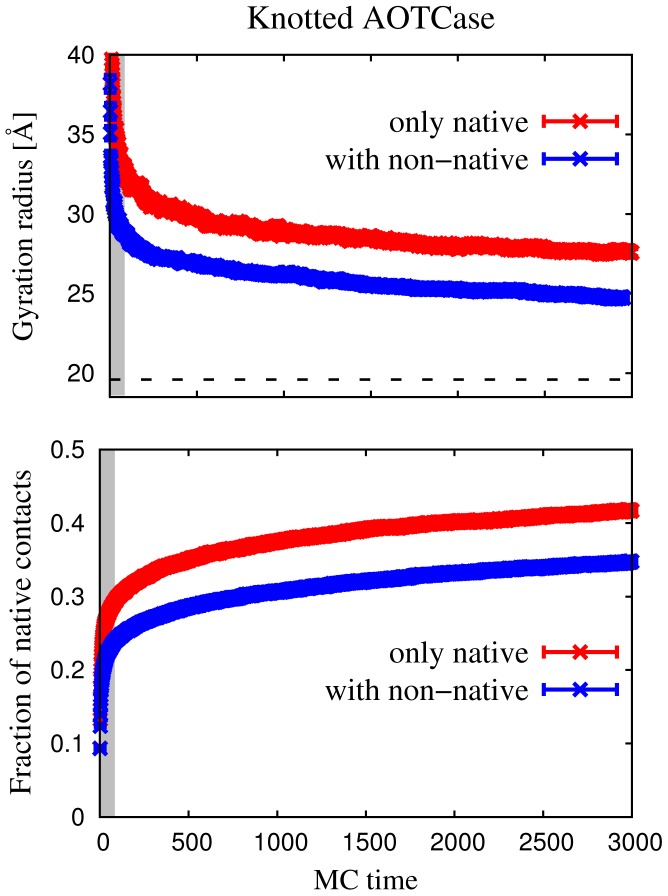
Monte Carlo time evolution of the average gyration radius (top panel) and fraction of native contacts (bottom panel) for the knotted AOTCase. The data obtained with the purely native-centric model are shown in red while those obtained with the added non-native interactions are shown in blue. In the top panel, the dashed line indicates gyration radius in the native state. Here, and in subsequent related figures, data points represent an average over the 150 trajectories and the associated statistical uncertainty is represented by the spread of the curves on the coordinate axis. One unit of MC time corresponds to 100 attempted MC moves per amino acid. The gray region denotes the initial MC evolution where global pivot moves are employed to relax the initial fully-extended conformation.

The folding dynamics is simulated using the stochastic Monte Carlo (MC) approach introduced and validated in Ref. [41]. The MC evolution entails a brief relaxation from the initial fully-extended state using both local and non-local moves. After this stage, indicated with a shaded region in Fig. 2 and related ones, the MC dynamics proceeds exclusively through local moves. The moves amplitude is sufficiently small that no chain crossings occurs, as verified *a posteriori. The stochastic scheme is analogous to the kink-jump dynamics* [*42*] *which, by virtue of the local character of the moves, is known to provide a physically-viable description of biopolymers' kinetics in thermal equilibrium* [*41]*, [43] *.*

The folding simulations are carried out using two alternative energy functions. The first one promotes exclusively the formation of native contacts between pairs of amino acids. Following Refs. [44], [45], the strength of the attractive interaction between any given native amino acid pair is derived from the strength of their hydrogen bonding in the native state and is expressed in thermal units, 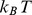, at the nominal Monte Carlo temperature of 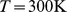. The second energy function includes electrostatic and non-native pairwise interactions in addition to the native-centric potential. The relative strength of the non-native interactions is set according to the quasi-chemical potentials of Miyazawa and Jernigan (MJ) [46] that reflect the statistical propensity of amino acid pairs to be in contact in proteins' native states. Following, again, Ref.s [44], [45], the average strength of the added non-native interactions is set to one tenth of the native one. No change to the MC temperature was made using this second potential because previous studies have shown that the addition of non-native interactions modifies the effective temperature of the system by less than 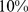 [47]. Consistently with this fact we have verified that the native states of both AOTCase and OTCase remain stable when evolved with the MC scheme using the second type of potential. In fact, the asymptotic value for average fraction of native contacts was 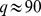
%. The fact that this value is smaller than the asymptotic one, 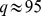
%, of the first type of potential indicates that the strength non-native interactions while weak enough to maintain stable the native state, can nevertheless compete with the native attractive interactions. The addition of the quasi-chemical interactions are therefore expected to be capable of accounting for sequence-specific non-native contact propensities in the partly-folded state. The sequence-dependent character of the non-native interaction differentiates the present approach from the early one of Wallin *et al.* [35] where the knot-promoting effect of non-native interactions was probed by systematically introducing attractive interactions between various pairs of segments of YibK.

**Figure 3:**
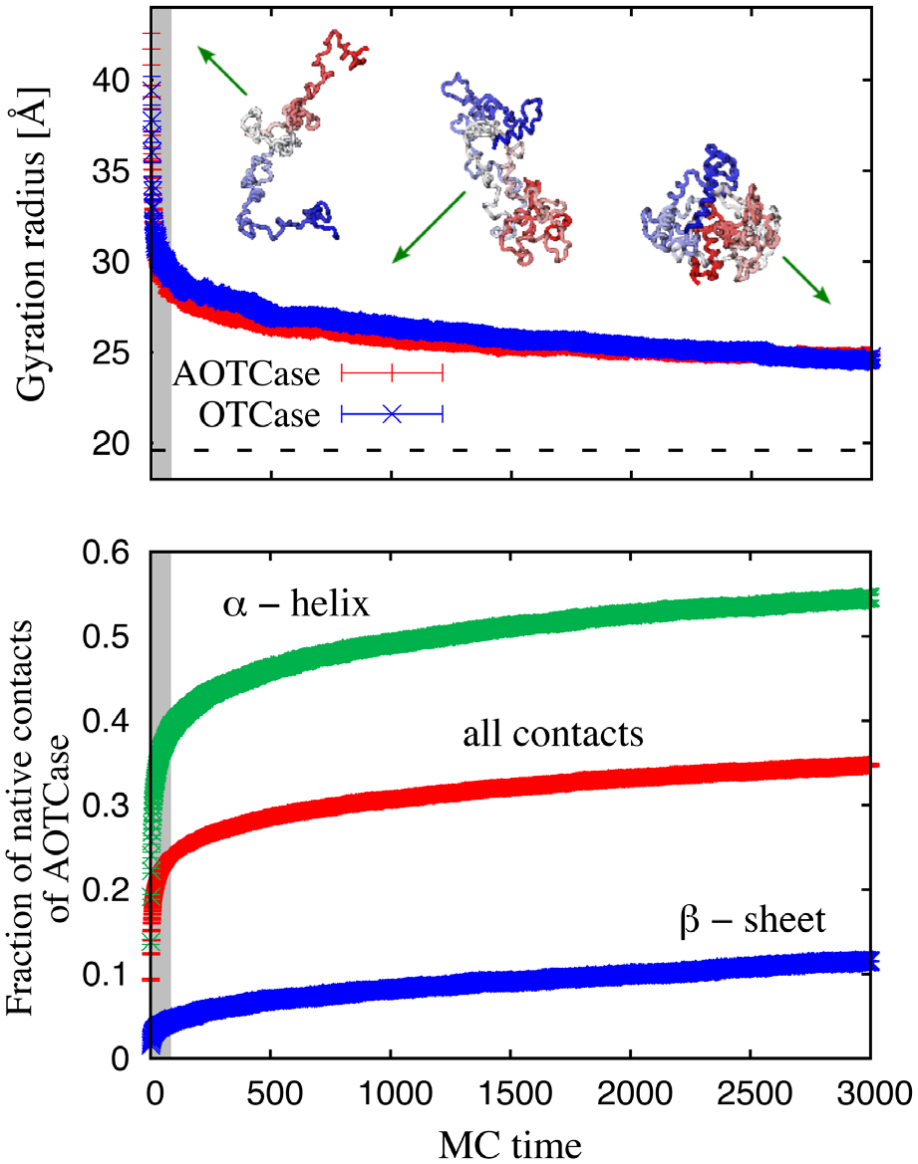
Top panel: Monte Carlo time evolution of the average gyration radius of the knotted AOTCase (red) and the unknotted OTCase (blue) in the model including both native and non-native interactions. The dashed line indicates gyration radius in the native state. Each data point is an average over the 150 trajectories and its statistical uncertainty is represented by the spread of the curves on the coordinate axis. The overlaid structures are instantaneous C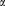 traces of AOTCase; the N and C termini are colored in red and blue, respectively. Bottom panel: Monte Carlo evolution of the fraction of native contacts of AOTCase. The overall fraction is shown in red, while the the fraction of formed native contacts involved in 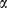-helices and 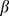-sheets are shown in green and blue, respectively.

For each of the two proteins and for each of the two energy functions, we generated 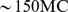 trajectories each consisting of 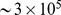 MC moves per amino acid (corresponding to 3000 units of MC time reported in Fig. 2 and Fig. 3), for a total of 75,000 CPU hours. The acceptance rate of the local MC moves for both proteins was nearly constant throughout the simulation and approximately equal to 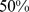.

The entanglement of the structures sampled during the MC evolution was established using a combination of two unrelated knot detection schemes, which are described in the Materials and Methods. This choice was made to maximize the robustness of the criterion used to establish the knotted state of an open chain - which is mathematically properly defined only after the chain termini are joined by a segment or arc thus giving a circular chain (chain closure operation). As described in the Materials and Methods section we used many alternative closures for each chain and adopted a majority rule to single out chains that have non trivial self-entanglements. These chains can correspond to proper, fully developed knots (fully accommodated in the knotted structures) as well as improper ones (partly accommodated in the closing arcs).

### Early folding kinetics

In order to illustrate how the folding process differs for the two potential energy functions, we show in Fig. 2 the evolution of the gyration radius (upper panel) and the fraction of native contacts (lower panel) of the knotted AOTCase, obtained by employing these two potentials. It is seen that the effect of the non-native and electrostatic interactions results in the higher compactness of the protein globule, since the gyration radius in this case lies systematically below the one calculated with the purely native-centric model. At any given stage of the MC evolution, the fraction of established native contacts is systematically lower for non-native interactions thus indicating that the latter introduce frustration that slows down the folding dynamics. Analogous results hold for the unknotted OTCase, see top panel of Fig. 3.

The data in Fig. 3 represent how the average radius of gyration and fraction of formed native contacts evolve in the course of the folding simulations of the two transcarbamylases. It is seen that, over the duration of the simulation, the average fraction of formed native contacts grows to 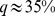
on average (the maximum value in all trajectories was 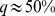. Overall, the probability of formation of native contacts decreases with the sequence separation of the amino acid pair, so that native 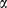-helical contacts are substantially more probable than inter-strand ones [48].

From the steady increase of the fraction of native contacts, 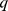, it is extrapolated that folding completion would occur on time-scales at least ten times larger than considered here. From the same figure it emerges that the radius of gyration gradually decreases to about 25 Å, which is 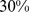 larger than the native one, consistently with the less compact character of the partially folded states.

To characterize the overall propensity of the two proteins to form knots while still largely unfolded, we extracted configurations at regular intervals of the simulations and analyzed their topological state.

### Non-native interactions and self-entanglement

**Figure 4:**
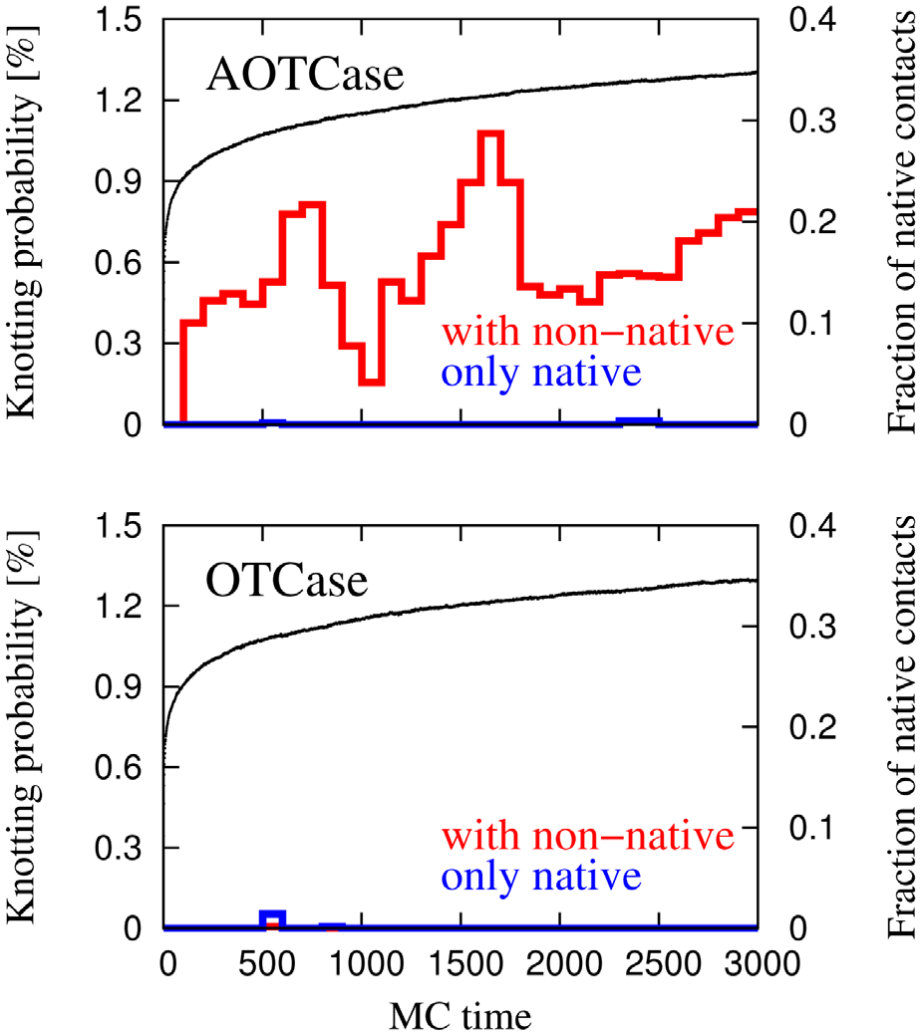
Monte Carlo time evolution of the average knotting probabilities and fraction of native contacts of the natively-knotted AOTCase (top panel) and of the unknotted OTCase (bottom panel). The thin black curve shows the average fraction of formed native contacts. The knotting probabilities observed for the purely native potential and for the added non-native interactions are shown with thick blue and red lines, respectively.

The knotting propensity of AOTCase, which is natively knotted, is illustrated in the upper panel of Fig. 4 which portrays the fraction of configurations that are properly or improperly knotted during a given time-window of the MC dynamics.

It is seen that when the purely native-centric potential is used, the knotting propensity is always negligible. However, when non-native interactions are added, the number of knotted configurations increases to a definite fraction of the total: at the end of the simulations, when only 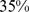 of the native contacts are formed, the fraction of conformations that are properly or improperly knotted is about 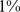. Notice that this value is comparable with the yield of purely native-centric coarse-grained models where pathways with a late-stage formation of knots are typically observed [36]. Within the limitations of coarse-grained approaches, the results indicate that sequence-dependent non-native interactions can produce a detectable fraction of configurations with the correct native entanglement already at early folding stages.

The analysis was repeated for the natively-unknotted OTCase and the results are shown in the lower panel of Fig. 4. The contrast with the case of the ATOCase is striking. In fact, the knotting probability is negligible not only for the purely-native case, but remains so even when non-native interactions are introduced.

The results indicate that the sequence-dependent non-native interactions – promoted by the quasi-chemical potential – increase dramatically the incidence of knots in the partially folded state of the AOTCase compared to the OTCase. Equivalently, the model calculations indicate that the two related natively-knotted and unknotted proteins have different knotting propensities already in the partially folded state, and that this difference can be ascribed to sequence-specific non-native interactions.

### AOTCase knotting events

To further elucidate the mechanisms responsible for these differences we monitored several parameters in the course of the simulations of the two transcarbamylases. More specifically, we searched for systematic differences in the interactions that the termini of these proteins establish with other parts of the peptide chain and for preferential locations of fully-developed knots.

**Figure 5:**
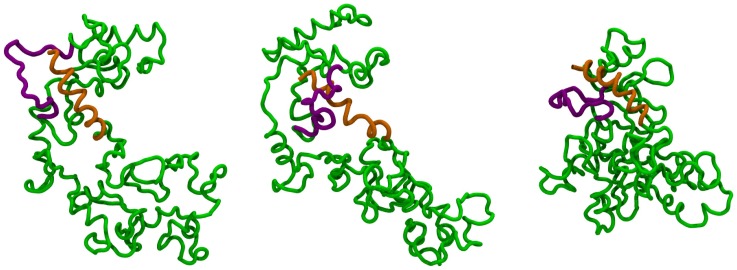
Three configurations of the knotted AOTCase in the presence of non-native interactions that illustrate a knotting-unknotting event. Three MC generated coarse-grained configurations (subsequent in MC time): unknotted (left and right) and knotted (middle). The knot results from the threading of a loop (in cyan) by part of the C-terminal 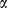-helix (colored in orange).

In this respect, it is important to point out that, in the course of the simulations for the AOTCase with non-native interactions, the process of knot formation is not irreversible. As illustrated in Fig. 5, trefoil knots can be formed and untied during each simulation. In particular, fully-developed knots can persists for up to one tenth of the duration of our simulations. Notably, only knots with the correct (i.e. native) chirality are observed.

By analysing the configurations preceding and following the knotting/unknotting events it is found that the change in topological state is typically caused by the threading of the helical C-terminus through loops formed by various protein regions.

Among the independent knotting events we identified six for which the stochastic closure returned a non-trivial topology in at least 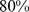 of the cases or the portion accommodating the knot had a depth of at least 20 amino acids. The analysis of the loop threading events leading to such persistent fully-developed knots showed that they involved the C terminus interacting with amino acids 55–65, 90–110, or 125–155. Only in one case it was observed the threading of the N-terminal through a loop involving segments 252–277.

**Figure 6:**
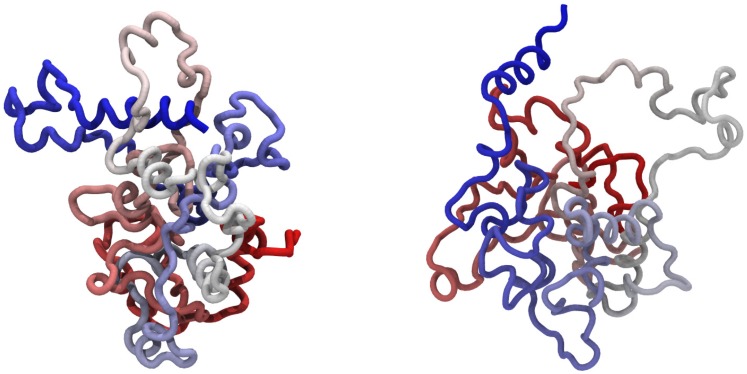
Typical protein configurations obtained in the presence of non-native interactions: that of the knotted AOTCase (left panel) and of the unknotted OTCase (right panel). The structures illustrate the tendency of the C-terminal 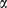-helix (colored in blue) to point towards the protein globule for the knotted protein and away from it for the unknotted one.

Representative configurations are shown in the left panel of Fig. 6 where it is clear that the helical C-terminus of the AOTCase typically points towards the rest of the protein, and the stiffness of the helix facilitates the threading of various loop regions.

At variance with the above situation, for both potential energy functions the OTCase C-terminus is typically exposed to the solvent and pointing away from the rest of the protein, as in the example shown in the right panel of Fig. 6. The terminus also shows a lower propensity to form 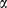-helices. An analogous situation is found for the AOTCase in the presence of only native interactions.

**Figure 7:**
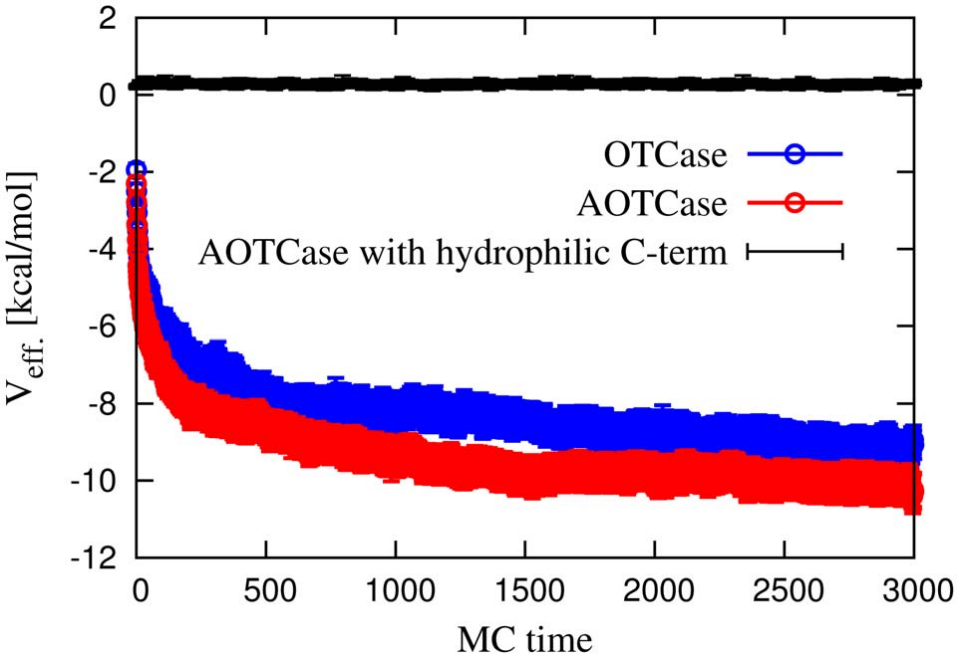
Monte Carlo time evolution of the average quasi-chemical interaction energy between the 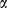-helix and the rest of the chain, in the knotted AOTCase and unknotted OTCase. The upper curve shows the same quantity for a mutant of the natively knotted protein in which all residues in the C-terminal 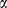-helix are replaced by hydrophilic residues.

The effect is quantitatively illustrated in Fig. 7, which portrays the average strength of the non-native interaction potential energy between the amino acids in the C-terminal 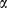-helix of the AOTCase or the OTCase and all the other residues in the chain. It is seen that the average non-native attraction is consistently stronger in the case of the knotted protein by about 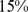, which is a sizable amount considering that the average is taken over all sampled structures, irrespective of their compactness and knotted topology.

The same figure also illustrates that, if the amino acids in the C-terminal 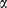-helix of protein AOTCase are replaced by neutral hydrophilic residues (GLU, GLN, ASN), then the average non-native interaction energy becomes slightly repulsive, explaining the tendency of the 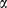-helix to point outwards. This provides an additional argument in support of the picture where the hydrophobicity character of the residues plays a role in the folding of knotted proteins [35].

**Figure 8:**
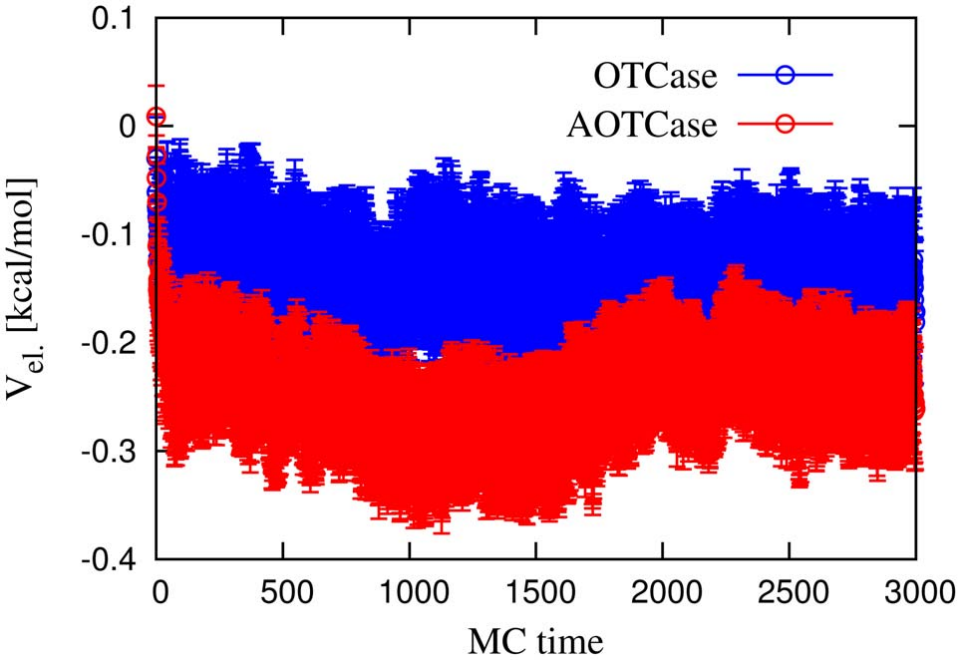
Monte Carlo time evolution of the average electrostatic interaction energy between the 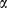-helix and the rest of the chain, in the knotted AOTCase and unknotted OTCase.

Finally, in Fig. 8 we plot the average electrostatic potential energy between the 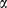-helix and the rest of the chain and show that, overall, the electrostatic interaction is about two orders of magnitude smaller than that of the quasi-chemical potential.

**Figure 9:**
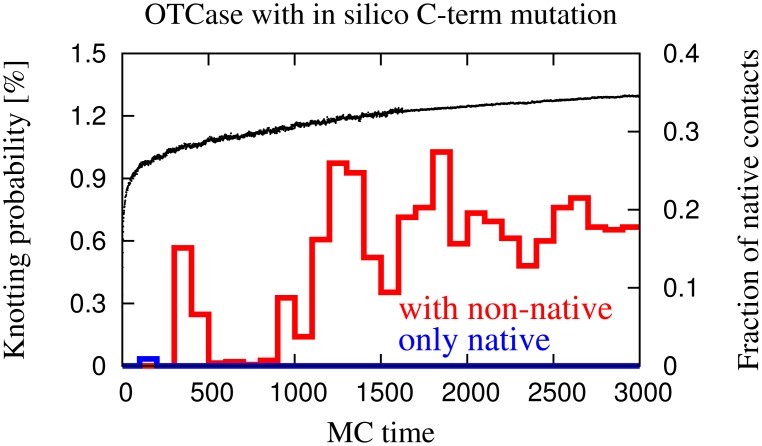
Monte Carlo time evolution of the average knotting probabilities and fraction of native contacts of the “in silico” mutant of the natively-unknotted OTCase. The knotting probabilities observed with the purely native-centric model and with the added non-native interactions are shown in blue and red, respectively. The black curve shows the fraction of native contacts in the presence of non-native interactions.

To further clarify the role of the C-terminal 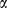-helix in the knotting of the partially-folded states of the AOTCase, we have carried out another set of simulations on a mutant protein. The last 25 residues of the OTCase (which are involved in the C-terminal 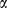-helix) were substituted with the same number of residues that form the C-terminal 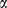-helix in the knotted AOTCase. Apart from such a substitution, all other attributes, namely the length of the chain and the native contact map, were identical to the unknotted protein. The outcome of the simulation is summarized in Fig. 9, from which we can conclude that the mutant protein forms a substantial fraction of knotted configurations in the presence of non-native interactions in its early stage of the folding.

### Summary and conclusions

A simplified coarse-grained protein model was used for a comparative study of early folding stages of two evolutionarily related transcarbamylase proteins: an AOTCase (PDB id 2g68) and an OTCase (PDB id 1pvv). The two proteins are well-alignable in sequence and structure and yet possess differently knotted native states: AOTCase is trefoil-knotted while OTCase is unknotted.

The role of sequence-dependent non-native interactions in promoting the correct native topology in the early folding stages was investigated by using two different energy functions in the simulations: one with a purely native-centric potential energy ( favoring only native contact interaction) and one with the additional contribution of quasi-chemical and electrostatic non-native interactions.

We found that in the absence of quasi-chemical interactions, neither protein shows an appreciable propensity to self-entangle in the early folding stages. However, once non-native interactions are introduced, the natively-knotted AOTCase does show a strongly enhanced propensity to form proper and improper knots with the correct native topology and chirality. By contrast, the knot enhancement effect is completely absent in the natively-unknotted OTCase.

Inspection of the ensemble of folding trajectories of the two proteins suggest that knotting in the partly unfolded AOTCase mostly results from the approach of the hydrophobic C-terminal 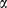-helix to other regions of the protein which are eventually threaded through. Because of the non-compact character of the partly-unfolded structures, the 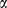-helix in the C-terminal can also retract from the threaded regions, so that various events of formation/disruption of proper and improper knots can be observed in a given trajectory.

The influence of the C-terminal region on the different knotting propensity of the two carbamylases is further supported by the folding simulations for an “in silico mutant” of the OTCase obtained by replacing the C-terminal sequence with the one of the knotted AOTCase. In fact, the folding simulations based on the quasi-chemical non-native interactions yielded a portion of knotted structures similar to the natively-knotted AOTCase, from which the C terminus was taken.

We recall that, the seminal study of Wallin *et al.* [35], which was based on a coarse-grained model of YibK with *ad hoc* non-native interactions, had suggested the relevance of non-native interactions for steering the early formation of the native knotted topology (by the threading of a loop by the C-terminal). Our results, which are consistent with these conclusions, provide additional elements to the picture by taking advantage of a coarse-grained framework where the folding of two carbamylases with similar structures, and yet different knotted topology are compared on equal footing. In particular our results indicate that non-native interaction propensities that are encoded in the primary sequence, can favor or disfavor the formation of knots already at early folding stages.

The necessarily limited scope of the coarse-grained approach used here does not clarify whether additional pathways leading to a late-stage formation of knots can be present in transcarbamylases. It would be most interesting to address this standing issue in future studies within the present, or alternative comparative schemes.

## Materials and Methods

### Coarse-Grained model

We have adopted the coarse-grained model developed in Ref.s [44], [45], in which the effective degrees of freedom are the amino acid residues, represented by a spherical bead located at the position of the corresponding 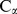 atom. The potential energy of the model consists of bonded and non-bonded terms. The bonded part of the of the potential consists of stretching potentials of the pseudo-bond 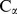-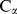 and pseudo-angle 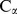-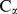-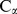, as well as potential for pseudo-torsions 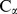-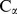-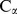-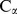:
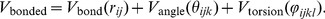
(1)

- The bond-stretching potential has the form
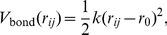
(2)where 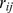 is the distance between the residues 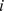 and 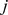, while 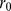 is the equilibrium length of the 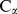-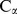 pseudo-bond.
- The double-well pseudo-angle potential is given by
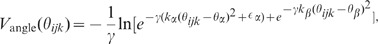
(3)where 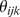 is the pseudo-angle formed by the residues 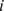, 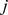 and 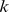, while 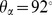 and 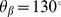 are the equilibrium values of the helical and the extended pseudo-angles, respectively.
- The torsion-angle potential for pseudo-torsions is of the form
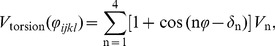
(4)with 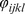 being dihedral angle between the planes (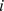,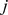,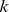) and (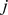,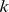,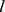). The constants 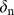 and 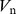 are depending only on the type of the middle two residues 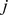 and 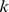 and are adopted from Karanicolas and Brooks [49].

All the values of the spring and energy constants appearing in the expressions of the bonded energy terms in Eqs. (2), (3) and (4) can be found in Ref. [45].

The non-bonded part of the potential consists of three terms, comprising native, non-native and electrostatic interactions, respectively:
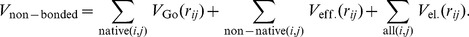
(5)

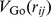
 denotes the G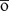-type potential developed by Karanicolas and Brooks [49]. Within such an approach, the native contact map is defined on the basis of the network of the hydrogen bonds in the native state, as well as on the degree of the proximity of the backbone atoms side-chains. Namely, two residues are defined to be in the native contact if the hydrogen bond between them is stronger than −0.5 kcal/mol, or if any of their non-hydrogen side-chain atoms are within the distance of 4.5 Å, in the native state. We do not consider contacts between residues with a distance in sequence smaller than 3 amino-acids. These interactions are described by the following functional form:
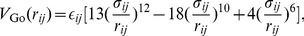
(6)where 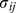 is the native-state separation of residues 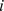 and 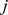. The strength of the interaction 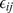 is chosen to be that of their hydrogen bond, for the residues that are hydrogen-bonded in the native state, while for the side-chain interacting residues it is a value proportional to the corresponding MJ contact potential [46] and it was suitably renormalized in order to match the hydrogen bond native contact energy scale. The residues in 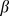-sheets or hairpins are often in contact via multiple hydrogen bonds, so in order to stabilize these structures within the G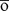-model, the additional network of weaker hydrogen bonds (having the strength 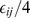) involving the neighboring pairs 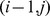, 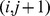, 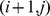 and 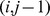 was introduced around each residue pair 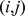 that was found to interact via either two hydrogen bonds or a hydrogen bond and a side-chain contact.
- Depending on the amino acid type of the residues 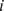 and 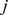, the non-native interactions in the model developed by Kim and Hummer in Ref. [45] can be both attractive and repulsive. Repulsive interactions are applied between amino acid pairs that interact less favorably with each other than with the solvent and *vice versa*. For a pair of residues that experiences an effective attractive interaction with a strength 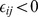, the non-native interaction potential is given by
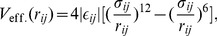
(7)with 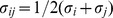, where 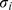 and 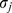 are van der Waals radii of the residues 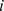 and 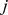.

For pairs of residues that effectively repel each other, so that 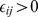, the non-native potential energy function takes the following form:
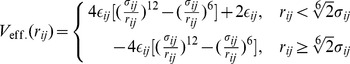
(8)


The effective Lennard-Jones interaction strength 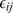 between residues 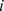 and 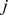 in the coarse-grained model of Kim and Hummer [45] is defined as
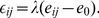
(9)The coefficients 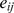 are negative and coincide with the entries of the MJ matrix [46], while 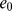 is an offset parameter. Hence, Eq. (8) defines a statistical knowledge-based potential which measures the preference of residue-residue interactions relative to residue-solvent interactions. The parameter 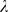 scales the strength of the Lennard-Jones interaction compared to the physical electrostatic interactions. The two free parameters 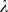 and 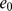 are fitted in order to correctly reproduce the binding affinity of the broad set of experimentally well-characterized protein complexes [45]. As an illustration, the effective interaction strength between the neutral hydrophilic residues GLU and GLN is 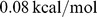, so that effective interaction is repulsive, while for the hydrophobic residues ILE and LEU is 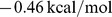, so that the effective interaction is attractive.

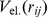
 is the long-range electrostatic interaction between residues 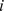 and 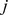 and it is modeled by Debye-Hückel-type of the potential
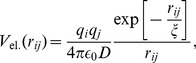
(10)where 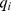 and 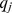 are the electrostatic charges of residues 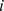 and 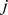, 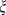 is the Debye screening length, 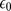 is dielectric vacuum constant and 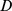 is the relative dielectric constant of water in near-ambient conditions.

### Numerical simulation details

The non-native interactions in the force field of our coarse-grained model introduce frustration and make the potential energy surface quite rugged. As a consequence, even within such a simple model, molecular dynamics (MD) simulations of the folding for chains of several hundreds amino acids are very expensive. Within the simulation time intervals which were accessible to our computer resources, such MD trajectories did not allow to monitor significant changes in the chain conformations.

To cope with this problem, we have simulated MC dynamics, using an algorithm which combines different types of moves, namely:

- local crankshaft moves [50], that consist of the rotation of a randomly selected single bead around the axis defined by its nearest neighbors. The angle of the rotation was randomly selected in the interval 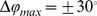,
- local end-point moves, in which the last 10 residues on both terminals are rotated rigidly with respect to the rest of the chain by up to 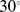 around a random axis passing through the most interior bead of the end segment.
- local Cartesian moves, that involve the displacement of the coordinates of a single randomly selected bead in the chain, within a sphere of radius 0.15Å,
- global pivot moves [51], where one amino acid is picked at random and the chain portion involving all amino acids with smaller (or alternatively larger) sequence index are rotated by up to 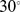 around a random axis passing through the picked amino acid.

The moves were accepted or rejected according to the standard Metropolis criterion.

MC algorithms based on local crankshaft and end moves are commonly employed in the polymer physics [41] to study *dynamic properties*, since it is was shown that they can mimic the intrinsic dynamics of a polymer in solution [50] at a much lower computational cost of MD simulations [52]. However, we emphasize that since our computational scheme is based on the MC evolution, we can not establish a quantitative mapping between the simulation time and physical time.

We have generated 150 independent MC trajectories for each system, starting from the same stretched coil configuration. In the early stage of the folding, these types of moves were attempted with the constant probabilities of 0.8, 0.1 and 0.1, respectively. Such an algorithm generates a rapid collapse of the chain from a fully stretched configuration to one in which the gyration radius is reduced from about 70Å to about 30Å in about 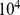 MC steps per particle, that corresponds to 100 units of MC time (see Fig. 3). During such collapse, no knot was observed. At the end of this stage, the acceptance rate of the global pivot moves typically dropped below 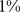. After this happened, we switched off the pivot moves, except from those involving few residues near the the two terminal of the chain. From this point on, the conformational changes of the chain are driven by the local crank-shaft and cartesian moves with an overall acceptance of about 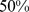.

When computing the fraction native contacts 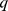 along the calculated MC trajectories, we adopt a criterion according to which two residues with index difference 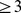 are said to be in contact if the distance of their 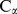 is less than 7.5Å.

### Knot detection schemes

The conformations visited during the Monte Carlo dynamics were topologically classified by computing the Alexander determinants after suitable closure into a ring [17].

For robustness, two alternative closure schemes are used: the minimally-interfering closure [53] and a modified version of the stochastic one [54]. The minimally-interfering closure is used first because it is very computationally effective, in that it entails a single, optimally chosen closure. In case of positive knot detection we further validate the non-trivial entanglement by performing 100 closures where each terminus is prolonged far out of the protein along a stochastically chosen direction, and the end of the prolonged segments are closed by an arc (that does not intersect the protein). The stochastic exit directions are picked uniformly among those that are not back-turning. Specifically, they must form an angle of more than 90 with the oriented segment going from each terminus to the 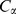 at a sequence distance of 10. If the majority of the stochastic closures return non-trivial Alexander determinants, than the conformation is non-trivially entangled. Such conformations can correspond to both proper, fully developed knots, and improper ones. The two can be distinguished using knot localization criteria [53], [55]: proper knots are entirely accommodated within the original protein chain, while improper ones span the exit segments. The knot type was determined using the scheme of Ref. [15], which is based on the KNOTFIND algorithm.
